# The Immunology of Zoonotic Infection

**DOI:** 10.3390/tropicalmed7070127

**Published:** 2022-07-07

**Authors:** Yang Li, Qiuchun Li

**Affiliations:** 1 Key Laboratory of Prevention and Control of Biological Hazard Factors (Animal Origin) for Agri-Food Safety and Quality, Ministry of Agriculture of China, Yangzhou University, Yangzhou 225009, China; dx120190147@yzu.edu.cn; 2 Jiangsu Key Lab of Zoonosis/Jiangsu Co-Innovation Center for Prevention and Control of Important Animal Infectious Diseases and Zoonoses, Yangzhou University, Yangzhou 225009, China; 3 Joint International Research Laboratory of Agriculture and Agri-Product Safety, Yangzhou University, Yangzhou 225009, China

Zoonotic infection can threaten public health locally and globally. The majority of emerging infectious diseases reported globally are zoonoses. The COVID-19 pandemic is a recently identified globally zoonotic infection [1]. Until now, many pathogens from animals have been reported to cause human infections, such as SARS, pandemic influenza H1N1, avian influenza H5N1 and H7N9, Middle East respiratory syndrome coronavirus (MERS-CoV), and *Salmonella enterica* serovar Typhimurium monophasic variants. However, much remains to be learnt about the immune responses induced by these pathogens. 

To develop methods to prevent and control zoonotic infections, we must reveal the immune responses of zoonotic infections, especially for the emerging or re-emerging microbes. Although mouse or chicken models have been widely used to reveal the immune responses of infections, there are frequently many differences in the symptoms of diseases among natural, transmission, and human hosts. Therefore, understanding the immune response of zoonotic pathogens in both animal models and natural hosts is likely to contribute to the discovery of new immune mechanisms [2]. 

The innate immunity and adaptive immunity systems coordinate to control the zoonotic infections in the host. The rapid initiation of innate immunity acts against any infected pathogens through the recognition of molecules frequently expressed in pathogens (the so-called Pathogen-Associated Molecular Patterns-PAMP) by Pattern Recognition Receptors (PRRs) on the surface of macrophages, dendritic cells, and natural killer cells. The macrophages and dendritic cells constitute the center of innate and adaptive immunity. These efficient antigen-presenting cells can elicit effector T cell responses or regulatory T cell responses [3,4]. 

Vaccination remains an efficient method to prevent and control zoonotic infections in both domestic animals and humans. Therefore, it is necessary to develop novel, efficient, and safe vaccines to prevent the spread of zoonotic diseases. Furthermore, these vaccines should induce the hosts to produce long-term protective immunity against pathogens [5]. 

In this special issue entitled “The Immunology of Zoonotic infections”, we welcome the discoveries of immune responses induced by zoonotic diseases, their pathogenesis, and developed vaccines against these pathogens.
